# Management and Outcome of Pipkin Type I and Type II Femoral Head Fractures by Ganz Surgical Dislocation of the Hip

**DOI:** 10.7759/cureus.67707

**Published:** 2024-08-25

**Authors:** Rajib Sarkar, Samriddhi Sarkar, Sayantika Sarkar

**Affiliations:** 1 Orthopaedics, ICARE Institute of Medical Sciences and Research, Haldia, IND; 2 Orthopaedics, Mahatma Gandhi Medical College and Research Institute, Pondicherry, IND; 3 Emergency Medical Services, Sir H. N. Reliance Foundation Hospital and Research Centre, Mumbai, IND

**Keywords:** post traumatic arthritis (pta), heterotopic ossification (ho), avn of femoral head, ganz surgical dislocation of hip, pipkin fracture

## Abstract

Introduction

Femoral head fractures, specifically Pipkin Type I and Type II, are uncommon injuries often linked with posterior hip dislocations. Management strategies for these fractures range from conservative treatments to various surgical procedures, with open reduction and internal fixation (ORIF) being a notable option. The surgical approach for ORIF varies, and due to the rarity of the injury, a standardized management protocol is lacking. This study aims to evaluate the outcome of managing Pipkin Type I and Type II femoral head fractures using ORIF through Ganz surgical dislocation of the hip, assessing complications and analyzing the functional outcome by radiographic assessment and functional evaluation.

Methods

This is a retrospective descriptive study of managing six cases of Pipkin Type I and Type II femoral head fractures with ORIF through Ganz surgical dislocation of the hip. Follow-up periods ranged from 19 to 96 months, and outcomes were evaluated using Matta's criteria for radiographic assessment and the Modified Harris Hip Score for functional evaluation.

Results

Known complications such as avascular necrosis (AVN) of the femoral head, heterotopic ossification (HO), post-traumatic arthritis (PTA), non-union of trochanteric osteotomy, and fracture non-union were monitored. Results showed one case of AVN, which occurred in a case of delayed open surgery following a failed primary closed reduction. All trochanteric osteotomies and femoral head fractures healed appropriately. No instances of HO or PTA were observed, even in the patient with the longest follow-up of 96 months.

Discussion

Controversy still exists in management and outcome of femoral head fracture among closed reduction alone, excision and ORIF using different techniques and approaches. Ganz surgical dislocation of the hip offers 360-degree visualization of acetabulum and nearly 360-degree visualization of head femur and hence an ideal exposure for working on femoral head and acetabulum. The study concludes that ORIF of femoral head fractures using the Ganz surgical dislocation approach is a viable treatment option, offering satisfactory outcomes with a low complication rate. The absence of PTA in long-term follow-ups may be attributed to meticulous removal of loose bodies and precise congruent reduction and fixation of head fragments.

## Introduction

Femoral head fractures are rare injuries that typically occur with posterior and less frequently anterior dislocation of the hip. Due to the rarity of these fractures, few studies are available, and most have a small number of cases [1-7]. Hosny et al. in 2022 reported other studies of managing femoral head fractures by surgical hip dislocation till their own study of 18 cases. Birkett first described and documented femoral head fractures in 1869 during a post-mortem dissection [8]. Pipkin published a classification system for femoral head fractures in 1957, which remains widely recognized today [9]. Pipkin’s classification distinguishes fractures based on the location of the fragment relative to the fovea: Type I (caudal to the fovea) and Type II (cephalad to the fovea). When a femoral head fracture is associated with a fracture of the femoral neck, it is classified as Type III, and when associated with an acetabular fracture, it is classified as Type IV. However, the Pipkin classification does not encompass all types of bone fragments, particularly osteochondral fractures and impacted fractures, nor does it address fragment size and fracture line direction. Since Pipkin’s classification, various systems have been proposed to address the fragment location, size, type of injury, joint stability, and associated injuries, aiming to describe the injury in detail to determine treatment protocols and prognosticate outcomes. Notable systems include those by Brumback (1987) [10], Yoon (2001) [11], Chiron (2004) [12], and the AO/Orthopaedic Trauma Association's (OTA) classification system as reported by Marsh [13]. Despite these efforts, all classifications have limitations and lack uniform applicability [14]. The simplicity of Pipkin’s classification keeps it popular, especially for isolated fractures of the femoral head (Pipkin I & II), which we propose should be considered discrete injuries, evaluated on their own merits. Associated injuries such as femoral neck fractures or acetabular fractures have distinct sequelae and prognostic factors and should be discussed separately.

In our study, we focus on isolated femoral head fractures, specifically Pipkin Type I and II, treated with open reduction and internal fixation (ORIF) using the “Surgical dislocation of the adult hip”, technique described by Reinhold Ganz in 2001. Ganz's technique, based on a detailed anatomical study of femoral head vascular supply, combines elements of previously reported approaches and consists of an anterior dislocation through a posterior approach with a trochanteric flip osteotomy. This method preserves the external rotator muscles and protects the medial femoral circumflex artery (MFCA) [15]. 

Various treatment modalities for Pipkin I and II fractures have been described in the literature, closed reduction alone, excision of fragment, and ORIF [14]. Different approaches have been adopted for open techniques, anterior (Smith-Petersen [16], Watson-Jones [17], Hueter [18], posterior (Kocher-Langenbeck [19] and its modifications, Gibson [20] ), medial based on the Ludloff approach [21], further modified and popularised by Ferguson [22]. However, these approaches are associated with high complication rates [23]. Previous reports are debatable due to inadequate sample sizes and lack of validated data. Specific complications reported in the literature include avascular necrosis (AVN), heterotopic ossification (HO), post-traumatic arthritis (PTA), and varied functional outcomes [24]. 

Aims and objectives

Our aims were: 1. To evaluate the outcomes of managing Pipkin Type I and Type II femoral head fractures using ORIF through Ganz surgical dislocation of the hip; 2. To assess the complications associated with this surgical technique, including AVN, HO, and PTA, non-union of trochanteric osteotomy, and fracture non-union; 3. To analyze the functional outcomes using Matta's criteria for radiographic assessment and the Modified Harris Hip Score for functional evaluation.

## Materials and methods

Study design

This was a retrospective descriptive study.

Patient selection criteria

*Inclusion Criteria* 

Adults with Pipkin Type I or Type II fracture who are physically fit and have an active lifestyle, considering physiological age over chronological age, with no pre-existing arthritic or any disease condition of the hip joint were included in the study. *Exclusion Criteria*

The exclusion criteria for this study include individuals who are physically unfit and lead a sedentary lifestyle, particularly those with a pre-existing arthritic condition or any disease affecting the hip joint. Additionally, cases where an immediate total hip replacement (THR) was performed were excluded. Patients with a femoral head fracture that is so severely comminuted that ORIF could not be performed are also excluded. Furthermore, those with Pipkin Type III and Type IV fractures are not eligible for inclusion in the study.

Table 1 shows the demographic data, variables of cases, follow-up period, functional outcome, and complications. All six occurred with posterior hip dislocation with high-velocity trauma.

**Table 1:** Shows the demographic data, variables of cases, follow up period, functional outcome, complications. AVN, avascular necrosis; HO, heterotopic ossification, RTA, road traffic accident; THR, total hip replacement; #, fracture; +ve, positive; -ve, negative

Case	Age In years	Gender	Mode of trauma	Side	Type of fracture	Time interval between trauma & primary reduction	Type of fixation	Drilling test	Follow up period (months)	Modified Harris Hip Score	Complications AVN,HO, Non union of trochanteric osteotomy or fracture head,
Case 1	69	Male	RTA hit by a car while walking	Left	Pipkin II +ipsilateral # shaft femur	4 hours	4 mm titanium cancellous screw-countersunk	+ ve	96	Excellent	Nil
Case 2	42	Male	RTA Motor cycle	Left	Pipkin II	52 hours	4 mm titanium cancellous screw-countersunk	-ve	19	Poor	AVN femoral head with united fracture THR performed.
Case 3	32	Male	Dashboard injury	Right	Pipkin I	3 hours	4 mm titanium cancellous screw-countersunk	+ ve	51	Excellent	Nil
Case4	44	Male	RTA Motor cycle	Left	Pipkin I	5 hours	4 mm titanium cancellous screw-countersunk	+ ve	46	Excellent	Nil
Case 5	38	Female	Dashboard injury	Right	Pipkin I	4 hours	4 mm titanium cancellous screw-countersunk	+ ve	39	Good	Nil
Case 6	49	Male	Dashboard injury	Right	Pipkin II	5 hours	4 mm titanium cancellous screw-countersunk	+ ve	41	Excellent	Nil

**Figure 1:**
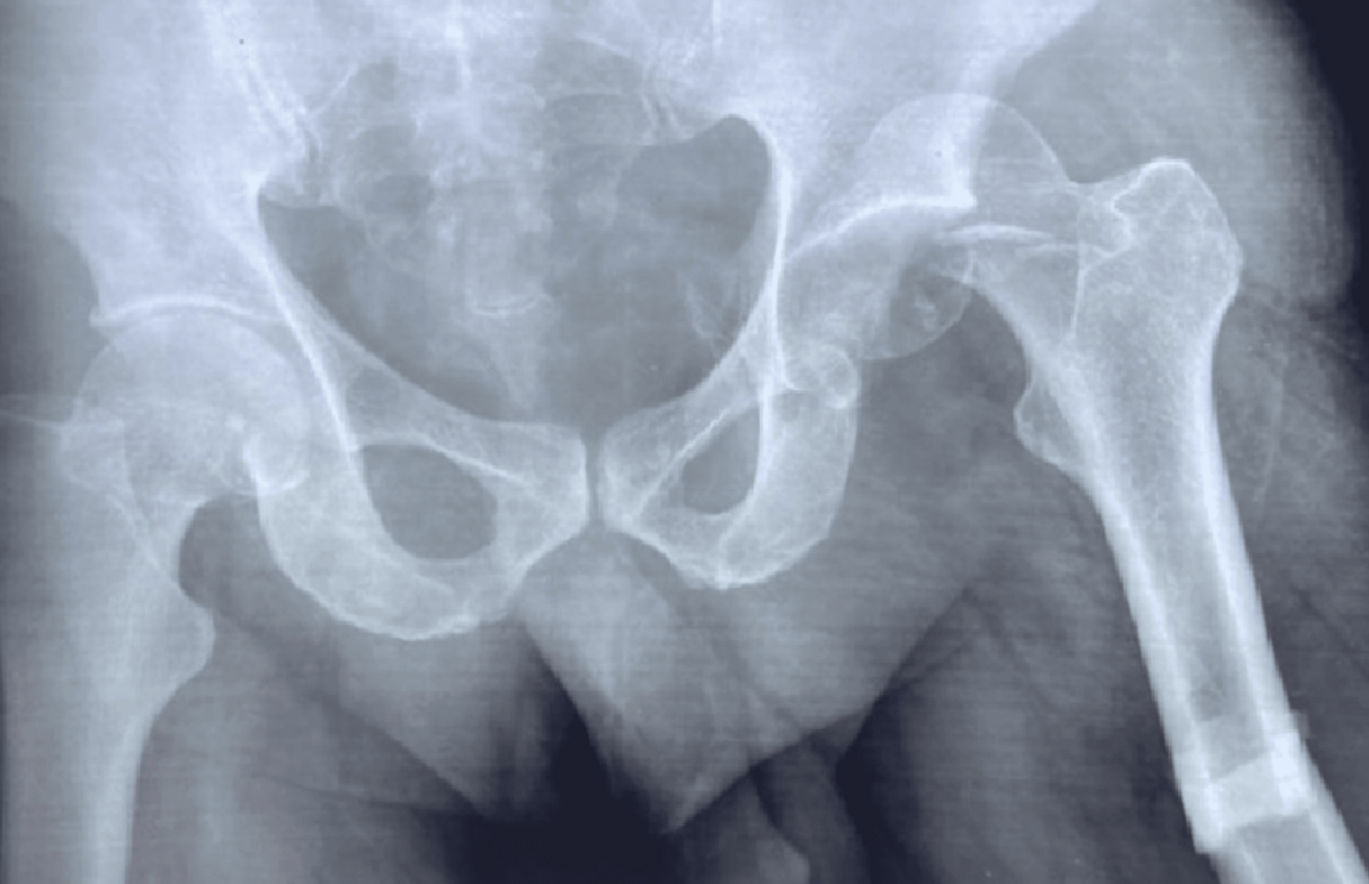
Pipkin Type II fracture left head femur with ipsilateral fracture shaft femur in a 69 years male (case no 1)

One, case no 1, with ipsilateral shaft femur fracture was haemodynamically unstable and received blood transfusion (Figure 1).

**Figure 2:**
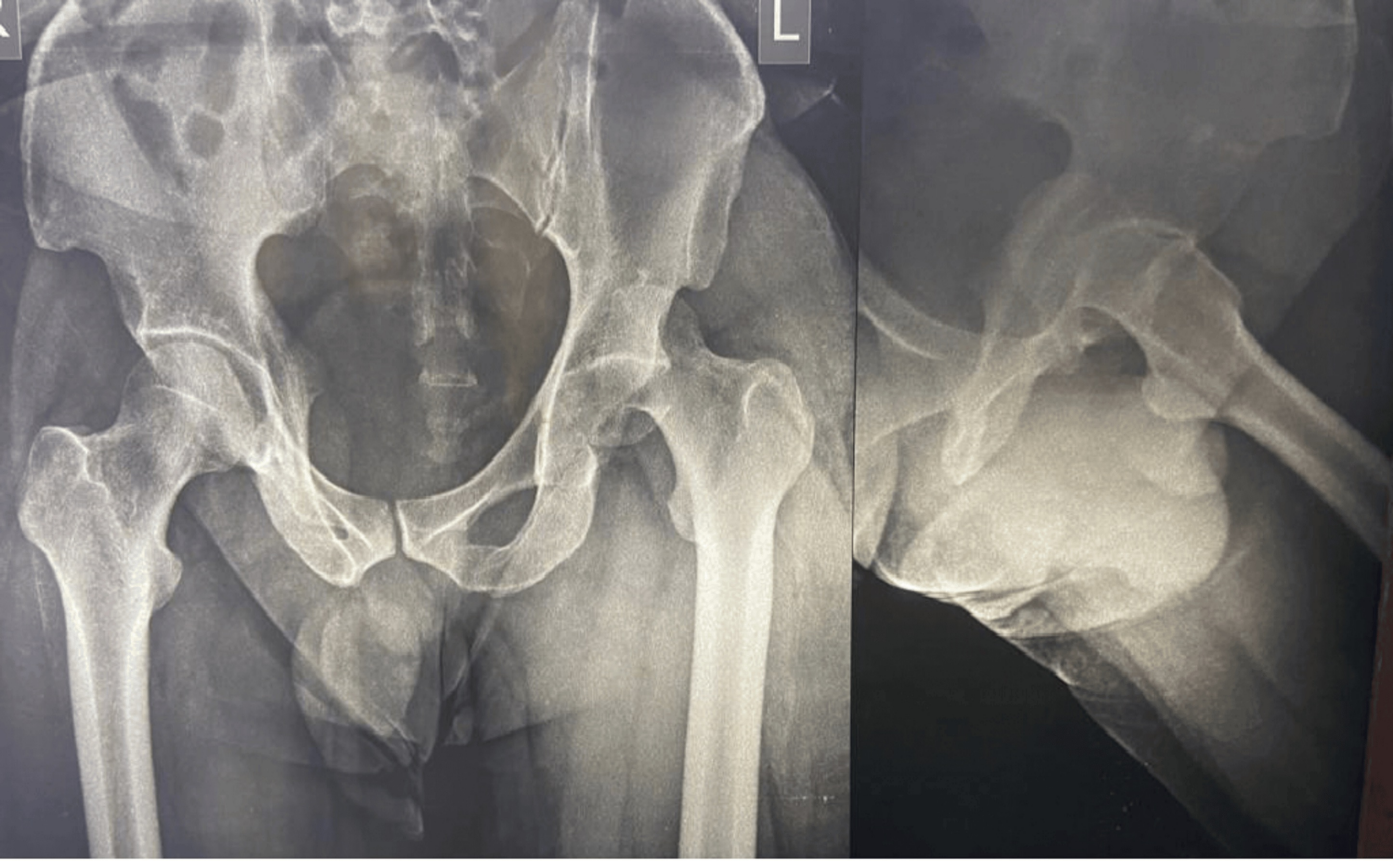
X-ray both hips anteroposterior (AP) and lateral (Lat) view of case no 2

**Figure 3:**
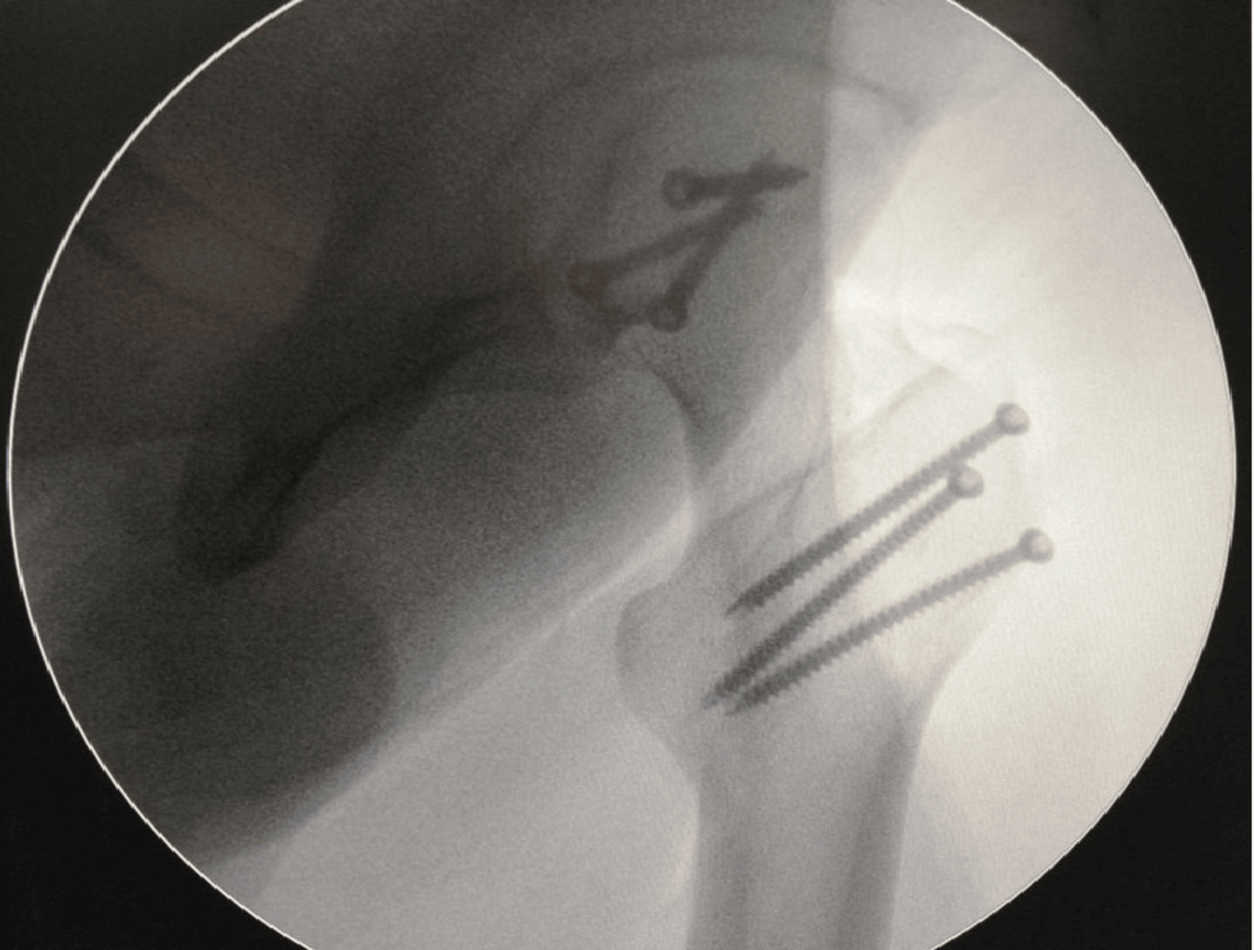
Immediate post operative C arm image

In five out of six cases, primary closed reduction succeeded within three to five hours of injury. In case no 2, closed reduction failed in the peripheral centre and was delayed by 52 hours before we performed immediate ORIF (Figures 2, 3).

**Figure 4:**
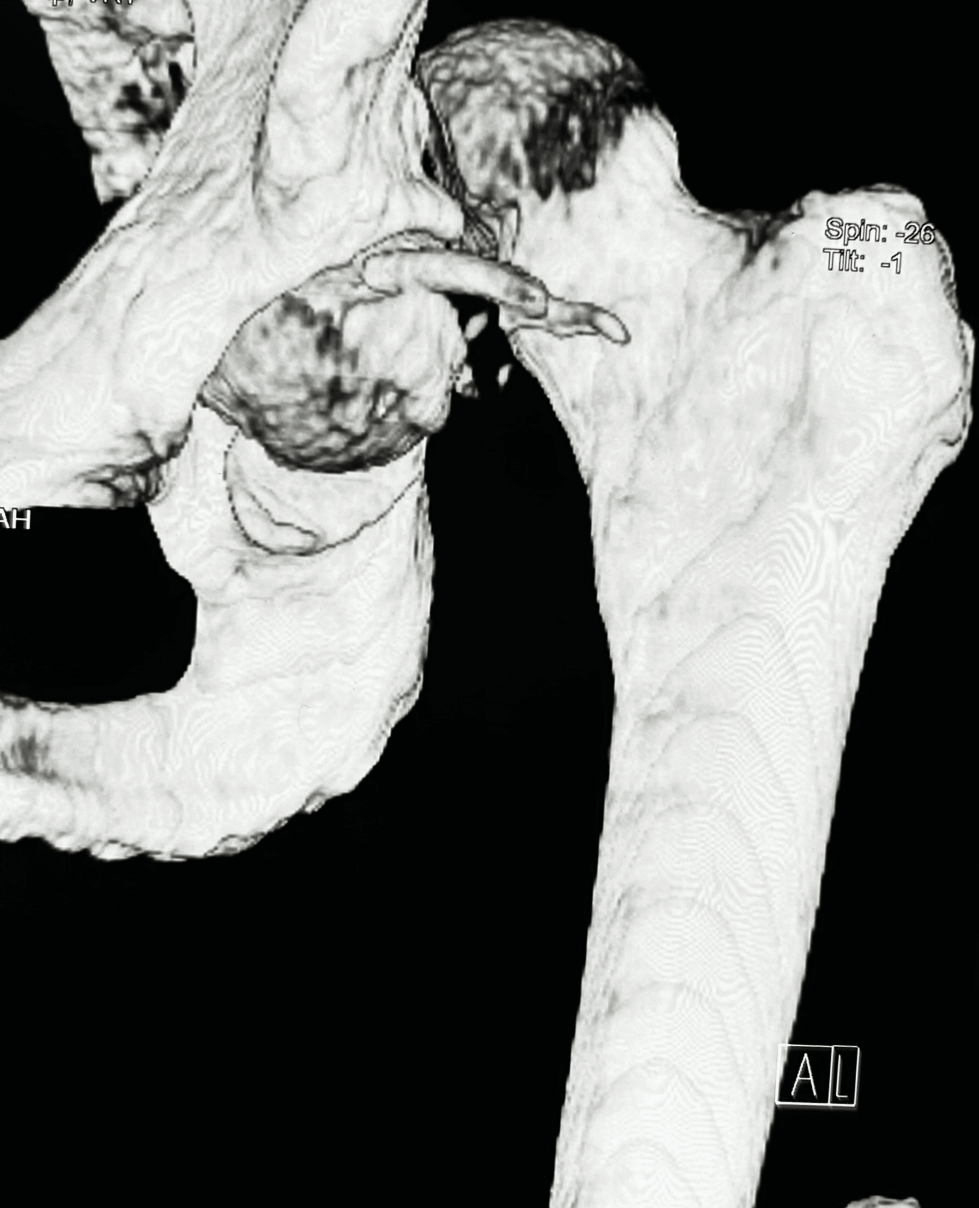
Computed tomography (CT) scan with 3D reconstruction delineating femoral head fragment size and multiple loose fragments of case no 1

All six cases had pre-reduction CT scan and post-reduction CT scans of all five cases. CT scans revealed the details of pattern of injury but missed minor chondral, osteochondral loose fragments, sandy particles, undermined cartilage and osteochondral fragments at fracture line as corroborated with subsequent per operative findings in all the cases (Figure 4).

Surgical technique

All the patients were operated in the lateral decubitus position under combined spinal-epidural anaesthesia. Kocher-Langenbeck incision was made. The fascia lata was incised and extended proximally by splitting gluteus maximus muscle. In only one case (case no 2) in which hip was unreduced, reduction was done at this stage by gentle digital palpation of dislocated head femur and doing some manoeuvre. In all the cases, no attempt was made to dissect and identify the posterior structures of hip. The posterior border of gluteus medius was identified. An incision was made from the posterosuperior edge of greater trochanter extending distally to the posterior border of the ridge of vastus lateralis.

Trochanteric Osteotomy

**Figure 5:**
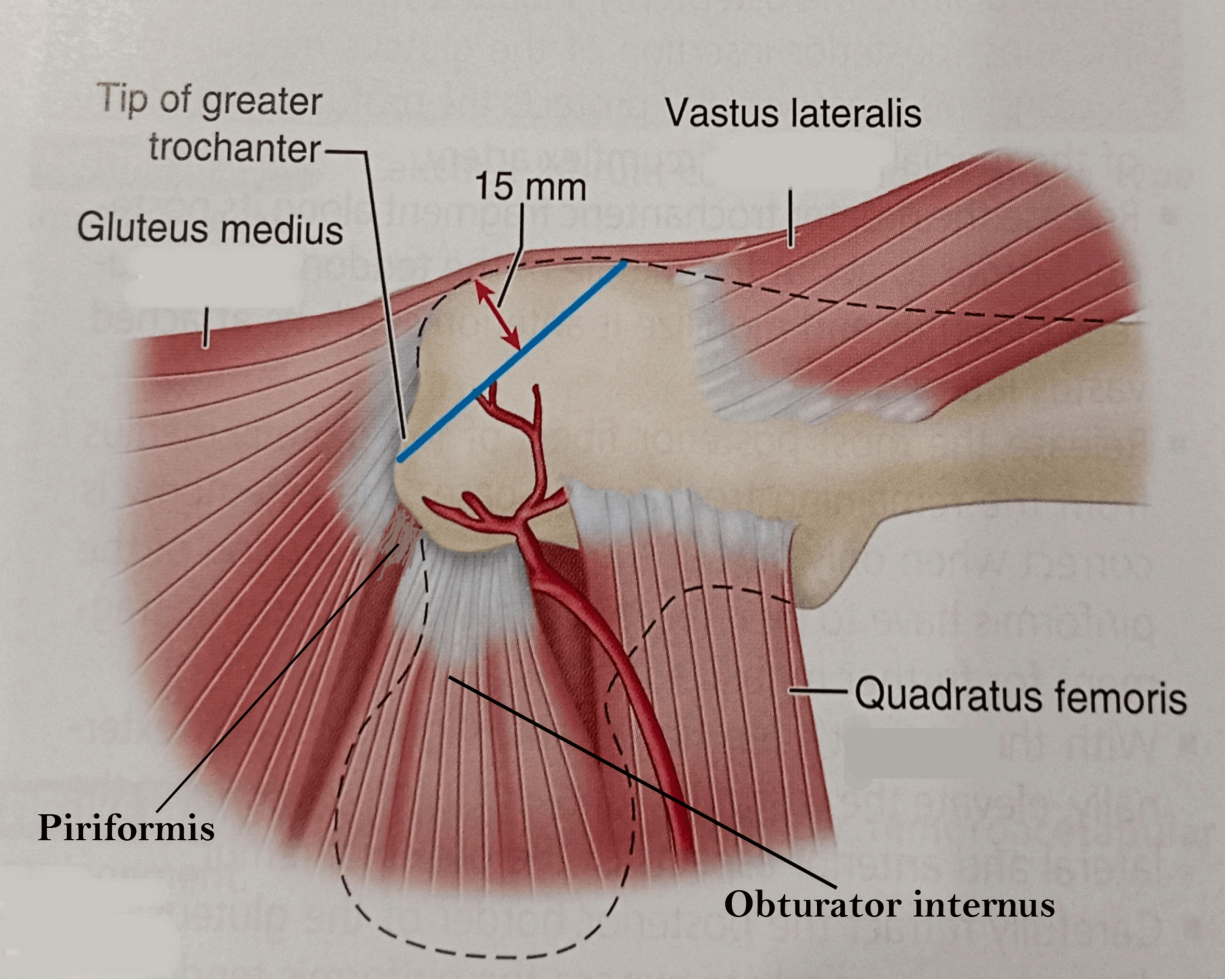
Line of trochanteric osteotomy image credit: Sandip Das, presented with permission

The osteotomy line was first marked with knife to create a 1.5 cm thick trochanteric flip. Multiple drill holes were made with a 2 mm drill bit at 2 cm intervals from posterior to anterior in a horizontal plane. The osteotomy is then completed with a power saw. Hence, a trochanteric osteotomy with a thickness of about 1.5 cm was made (Figure 5).

**Figure 6:**
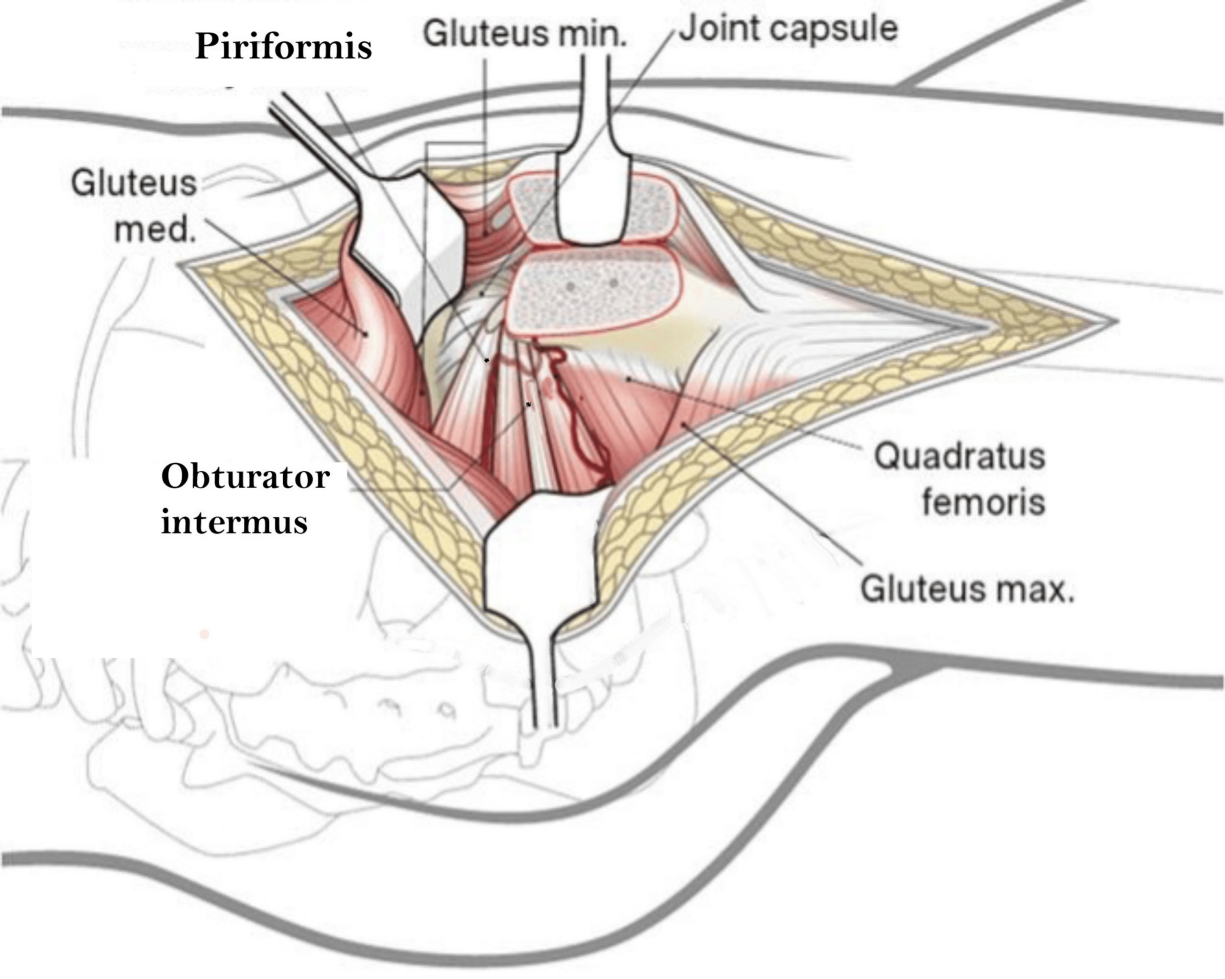
Digastric trochanteric flip osteotomy image credit: Sandip Das, presented with permission

The osteotomy exited just anterior to the most posterior insertion of gluteus medius, the fibres were released from the remaining trochanteric base. The trochanteric flip osteotomy fragment is being a digastric osteotomy having gluteus medius attachment proximally and vastus lateralis attachment distally, was mobilised anteriorly with its attached muscles. With the leg flexed and slightly rotated externally vastus lateralis and intermedius were elevated from the lateral and anterior aspects of the proximal femur. The inferior border of the gluteus minimus was separated from the relaxed pyriformis and underlying capsule. The anterior, superior and postero-superior capsule was visualised (Figure 6).

**Figure 7:**
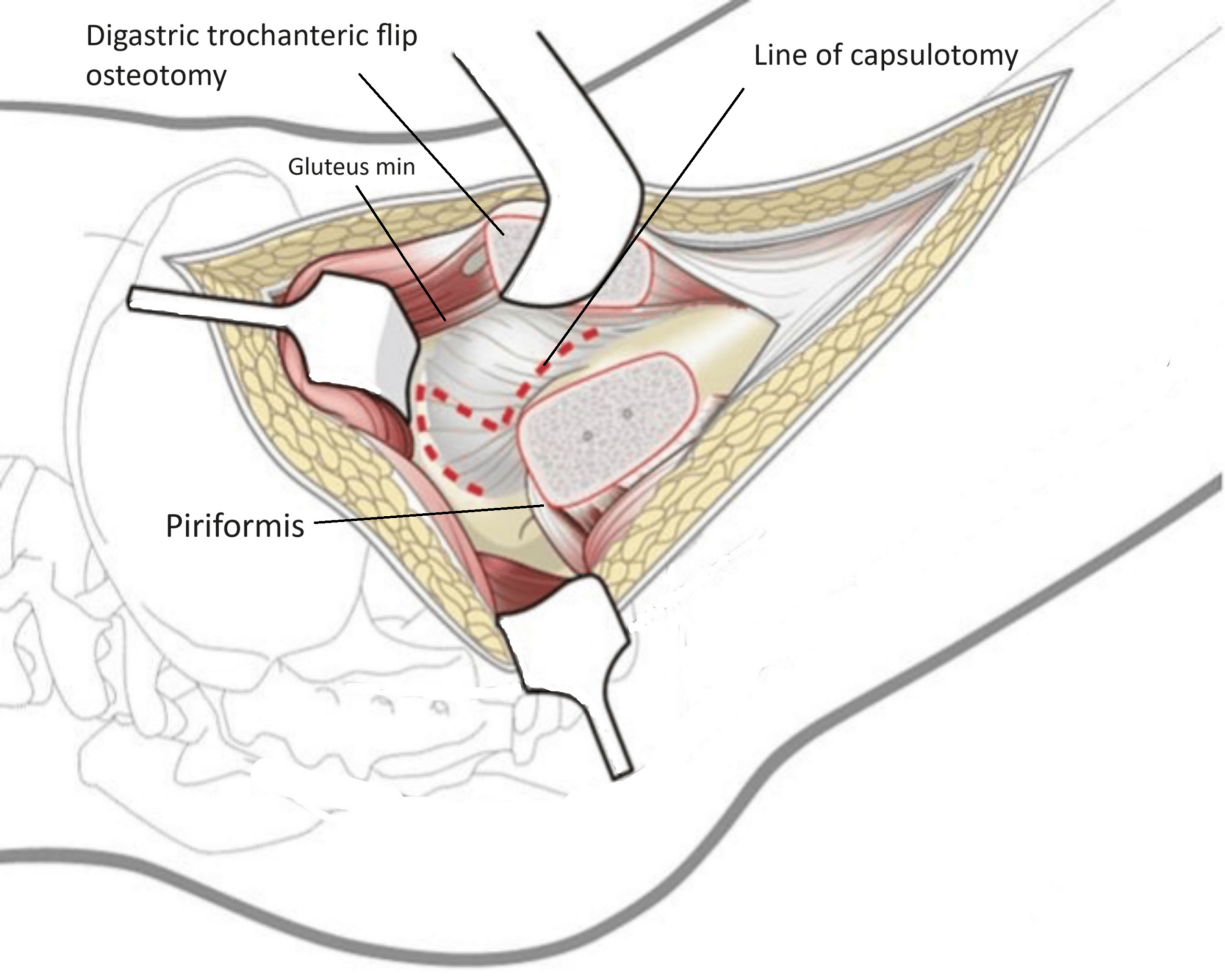
Line of capsulotomy image credit: Sandip Das, presented with permission

The capsule was first incised anterolaterally along the long axis of femoral neck from osteotomy towards the acetabulum. The second cut was along the distal anterior insertion of the capsule around the calcar. The third cut was parallel to the edge of the acetabulum in a posterior direction (Figure 7). When a traumatic tear is encountered it is carefully incorporated within the capsulotomy.

**Figure 8:**
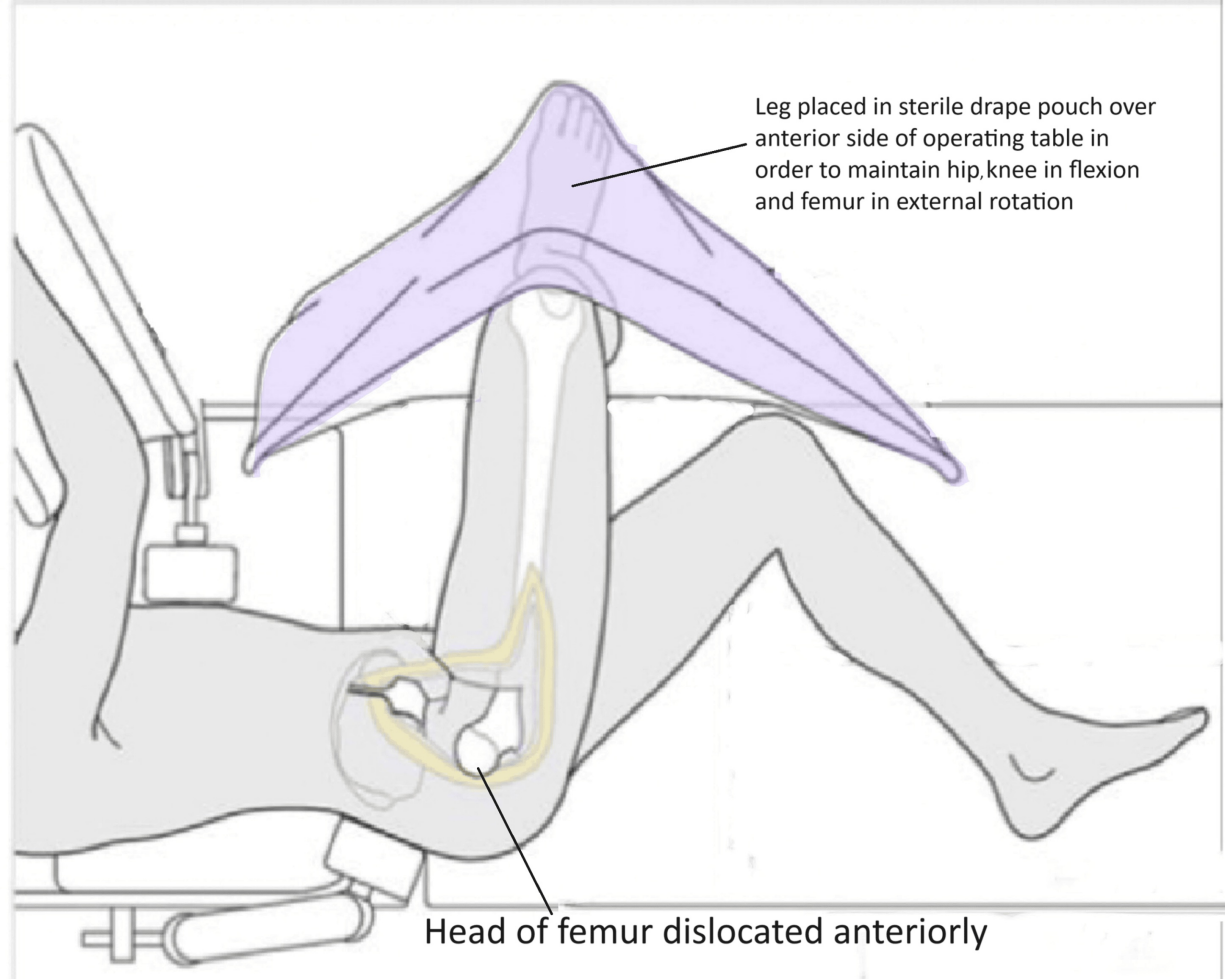
Position of patient and limb image credit: Sandip Das, presented with permission

Complete anterior dislocation was obtained through continuous traction with a flexed knee, progressive hip flexion and external rotation of the femur causing more subluxation to complete dislocation. Gentle pulling by a finger placed around calcar facilitated the dislocation, we avoided bone hook with assumption of causing damage to retinacular vessels. If the ligamentum teres was not torn as found in two of our cases, it was cut with long sharp curved scissors with great care to protect the medial retinaculum, attached to inferior fragment. The limb was put in a sterile side bag (Figure 8).

**Figure 9:**
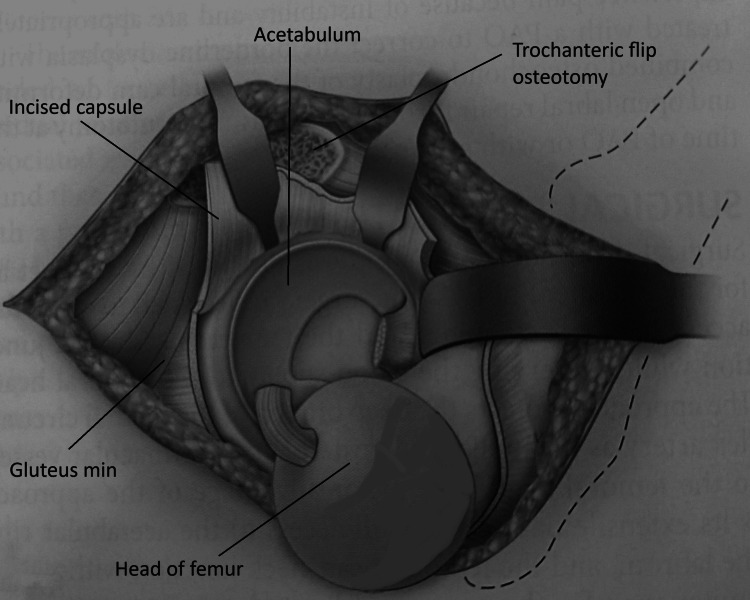
360-degree visualisation of acetabulum and head with surgical dislocation of hip image credit: Sandip Das, presented with permission

 After dislocation, 360-degree visualisation of acetabulum and femoral head was obtained (Figure 9).

**Figure 10:**
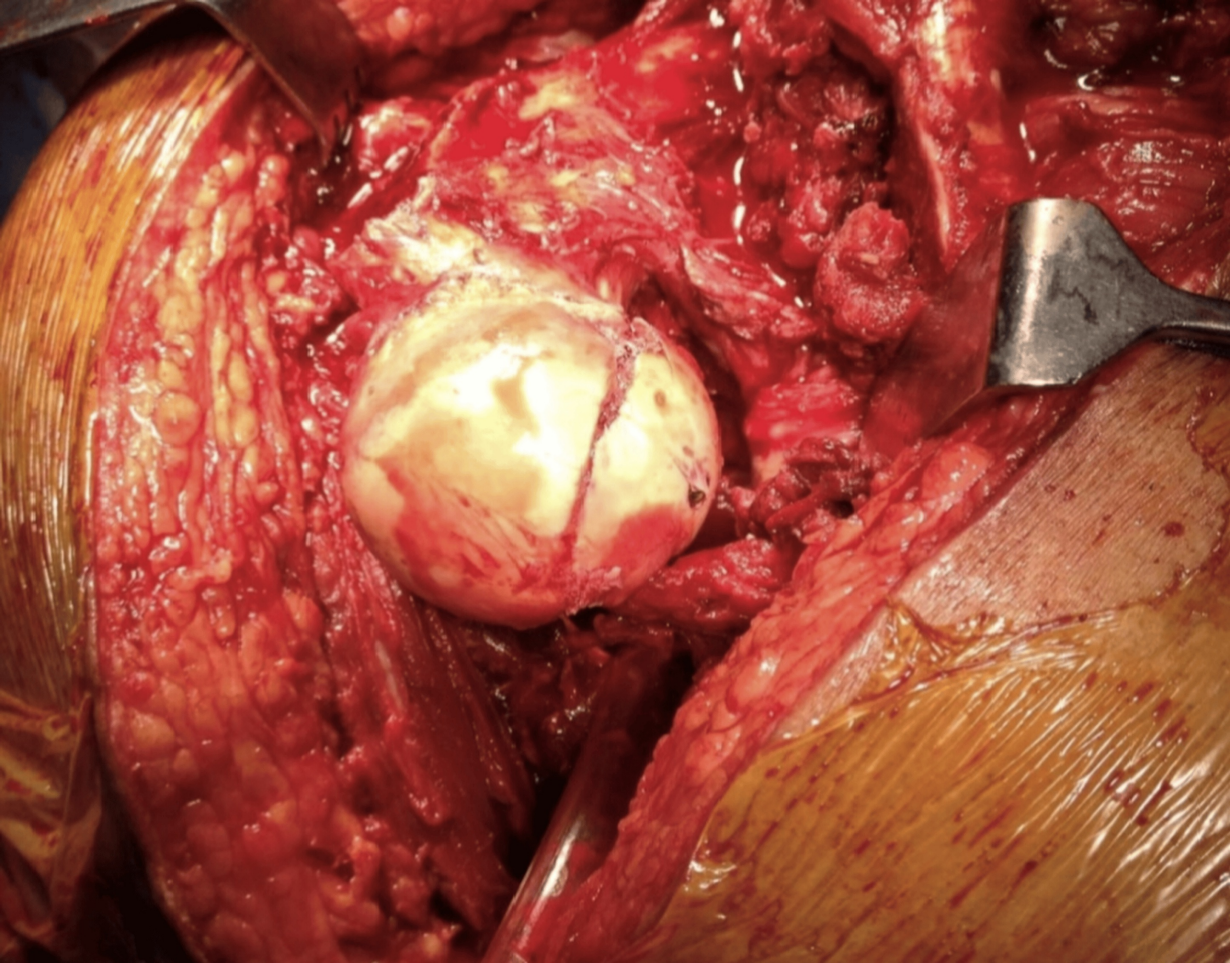
Open Reduction and Internal Fixation (ORIF) fracture head femur with three 4 mm titanium cancellous screws after debridement, removal of loose fragments and sandy particles by thorough lavage done with Ganz Surgical Dislocation of hip

Thorough exploration and observation were done, besides main fracture fragments of femoral head, multiple minor chondral, osteochondral and sandy particles were found in all the cases and most of which could not be detected in pre-operative CT scan. All loose bodies including sandy particles were removed by thorough irrigation. The fracture margins in all the head fragments were found frayed having loosely attached small osteochondral and purely chondral fragments which were undermined. All these unhealthy, partly loose fragments, undermined cartilage was debrided which resulted in some gap (crater) between fragments after reduction. Overall articular congruity and femoral head sphericity was strictly maintained in reduction with clear plastic spherometer, fixed provisionally with two to three K-wires, final fixation was done with three 4mm titanium cancellous screws with countersinking of heads. In no case the crater exceeded 5mm in either width or depth (Figure 10).

**Figure 11:**
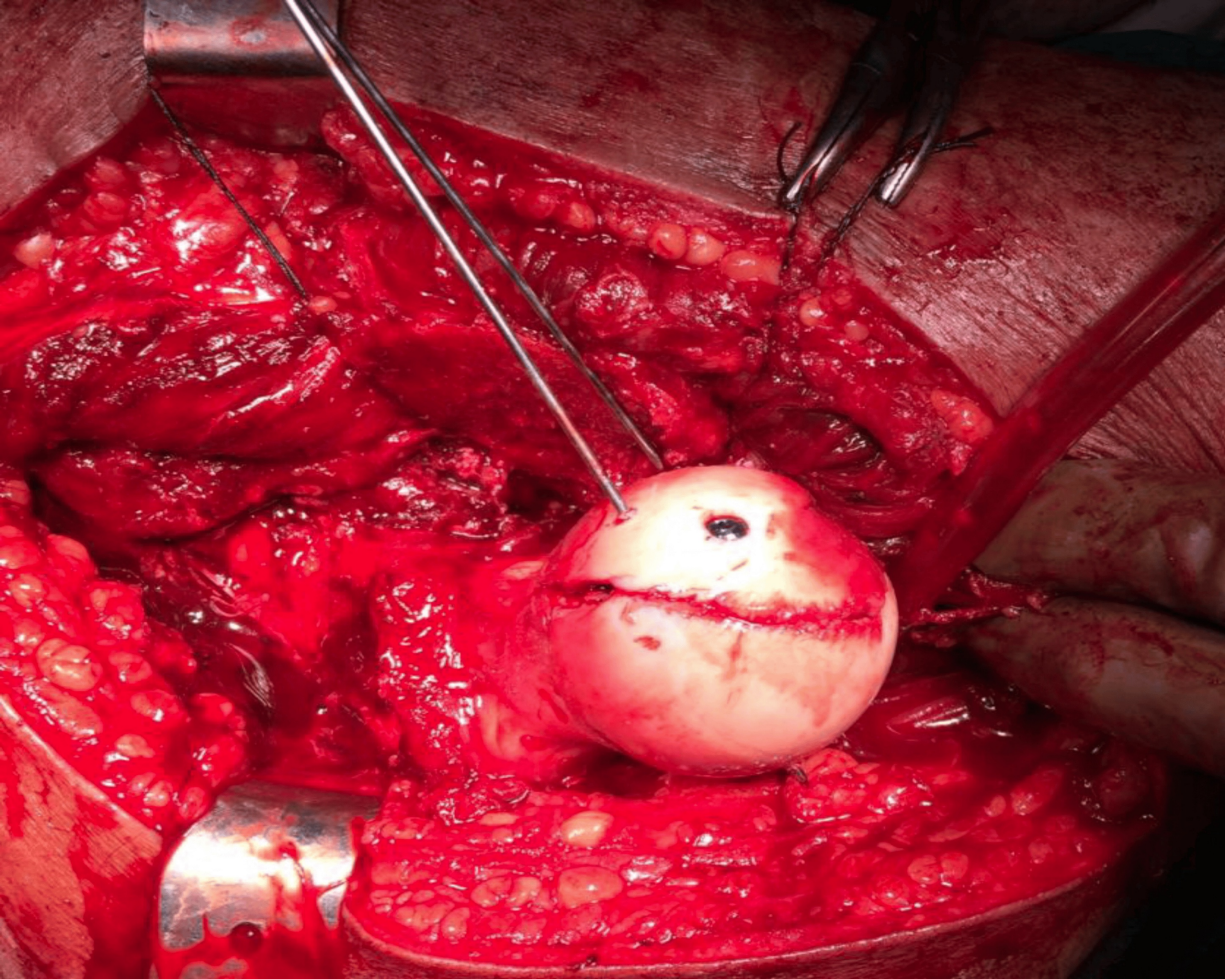
Per operative – Open Reduction and Internal Fixation (ORIF) – negative drilling test

In all the cases posterior acetabular labral tear and lacerations were found, which were debrided only. Apart from fracture margins, the rest of the head cartilage as well that of acetabulum looked healthy. The vascularity of the femoral head was checked by “Drilling Test” or “Bleeding Sign” done with a 2 mm drill bit. Active bleeding on drilling was appreciated in five out of six cases. In case 2 with delayed reduction of hip by 52 hours, the test was negative (Figure 11).

The hip joint was thoroughly irrigated again and gentle reduction of femoral head was done. A very specific ”Sound of sucked in” was appreciated with reduction of femoral head into acetabulum in all cases and a negative suction force was appreciated in an attempt of separating the head from acetabulum by manual traction. This was appreciated, in our opinion, by achievement of perfect articular congruity and sphericity of head resulting in congruous hip joint. The capsule was repaired without tension. Meticulous haemostasis was done throughout the procedure. The greater trochanter flip osteotomy was returned to its original position, accurately reduced and fixed with three 4mm titanium cancellous screws. Wound closed in layers leaving two separate negative suction drains, one anterior and one posterior, which were kept for 48 hours.

**Figure 12:**
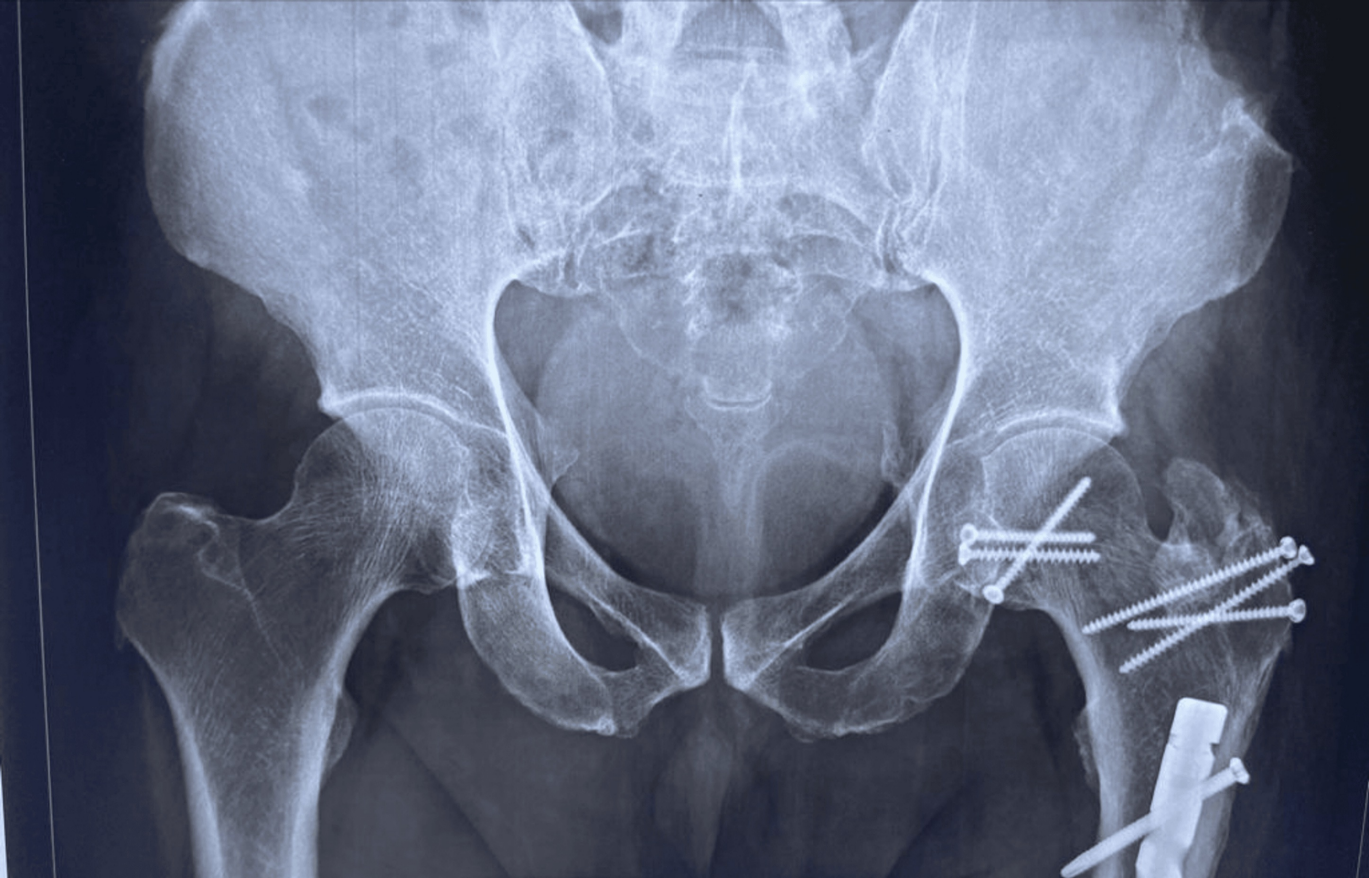
12 weeks follow-up, both trochanteric osteotomy and fracture head fragment united with interlocking nail for ipsilateral fracture shaft femur.

In one case (case 1) who had an associated ipsilateral fracture shaft femur, an interlocking nailing was done after trochanteric osteotomy, the nail was introduced beyond the osteotomy for subsequent fixation of trochanter, we proceeded for further steps for surgical dislocation of hip for dealing fracture head femur (Figure 12).

Post-operative protocol

All the six cases had no associated co-morbid conditions. Five out of six cases (except case 2) were put on enoxaparin 40 mg once daily, stopped 12 hours before surgery, resumed in all five cases and started in case 2 after two hours of removal of epidural cannula and wound suction drain, continued for three weeks till they were freely mobile on their own. As prophylaxis against HO, indomethacin 150 mg/day in three divided doses was given in all cases and continued for one month. All the patients were mobilised within bed on first postoperative day, standing and walking with partial weight bearing on second postoperative day. Postoperative mobilisation was supervised by a physiotherapist with gradual progression to independent full weight bearing achieved by three months. No case had postoperative infection.

To monitor the development of complications like AVN, HO, PTA, and union of osteotomy and femoral head fracture, the cases were followed up, besides peri operatively, at six weeks, 12 weeks, nine months, 12 months, and then annually or if a new complaint occurred.

Follow-ups included plain radiographs of both hips - anteroposterior and frog lateral views. Matta's criteria were used to assess reduction and union. Development of AVN, HO, and PTA were looked for in the periodical follow-up plain radiographs.

Functional evaluation

Modified Harris Hip Score was used to assess function at each follow-up at six weeks, 12 weeks, nine months, 12 months, and then annually. All follow-up assessments were done by the first author and approved by each of the co-authors independently.

## Results

In the series five are male and one is female. Three had left and three had right femoral head fracture. Mean age is 46 years with range 32 to 69 years. Mean follow-up is 49 months with range 19 to 96 months. The mode of trauma was motor car accident (dashboard injury) in three, motorcycle accident in two and hit by a highspeed car in one case, all had high-velocity injury. Three cases had Pipkin Type I and three had Type II fracture. One had associated ipsilateral femoral shaft fracture. Intraoperatively, all cases had minor chondral, osteochondral fragments and sandy particles. Fracture margins of all head fragments required debridement because of partly loose and undermined articular cartilage fragments. The details of minor loose particles of injury couldn’t be assessed by preoperative CT scan. Crater at fracture margins was upto 5 mm in width and depth.

**Figure 13:**
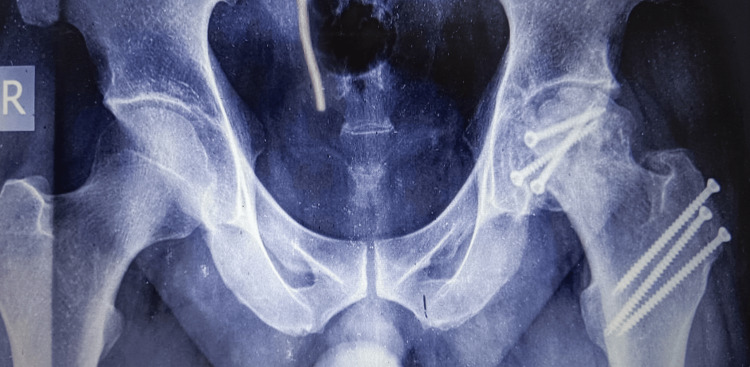
At 19 months follow-up – avascular necrosis (AVN) developed (case no 2)

**Figure 14:**
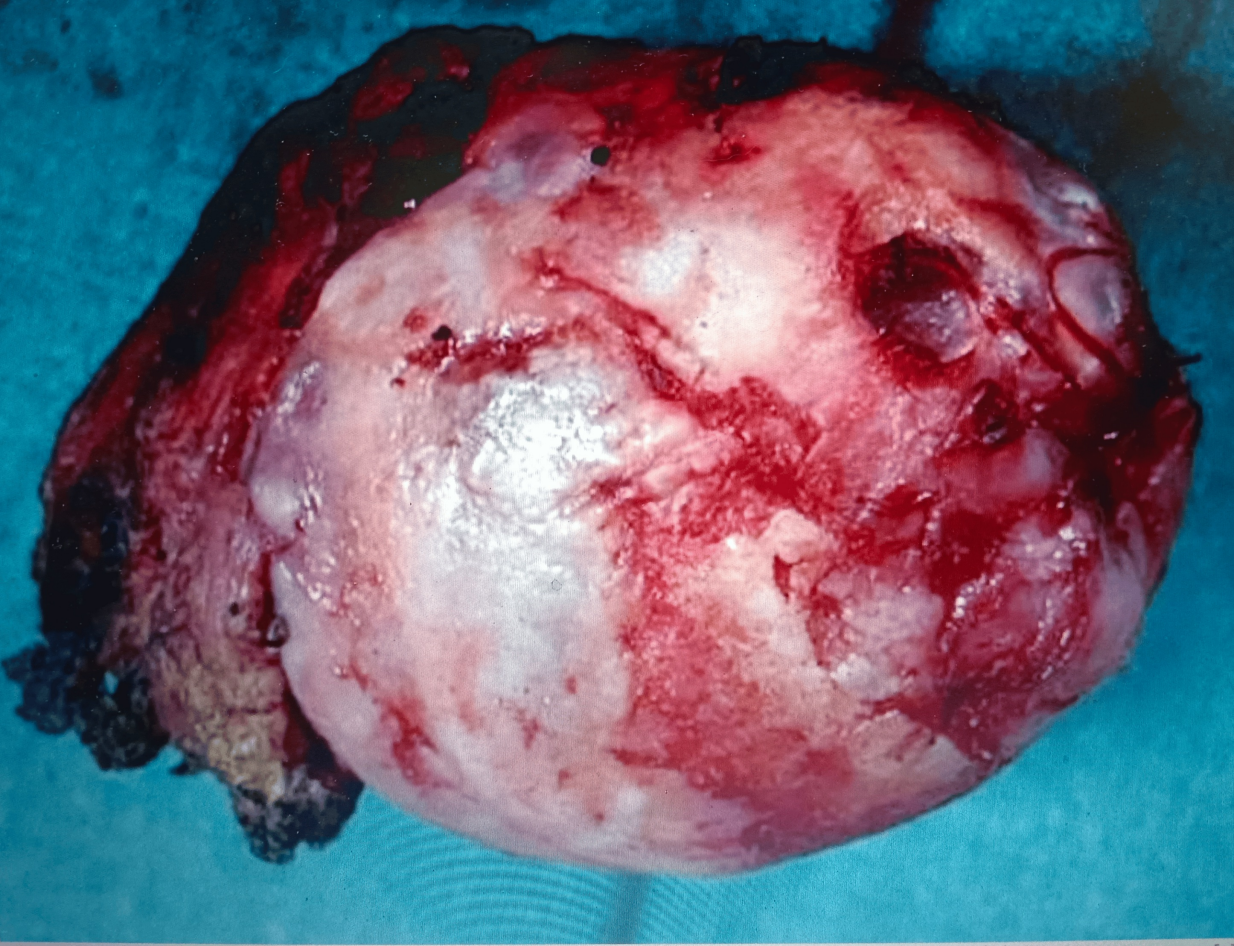
Excised head of femur of case no 2 – irregular articular surface with collapse as avascular necrosis (AVN) developed with union of fracture head femur

Closed reduction succeeded for five out of six cases within three to five hours of injury. In one case reduction failed in a peripheral centre and was delayed by 52 hours. This patient had negative drilling test and developed AVN femoral head with union of fracture head femur at 19 months follow-up period (Figures 13, 14).

**Figure 15:**
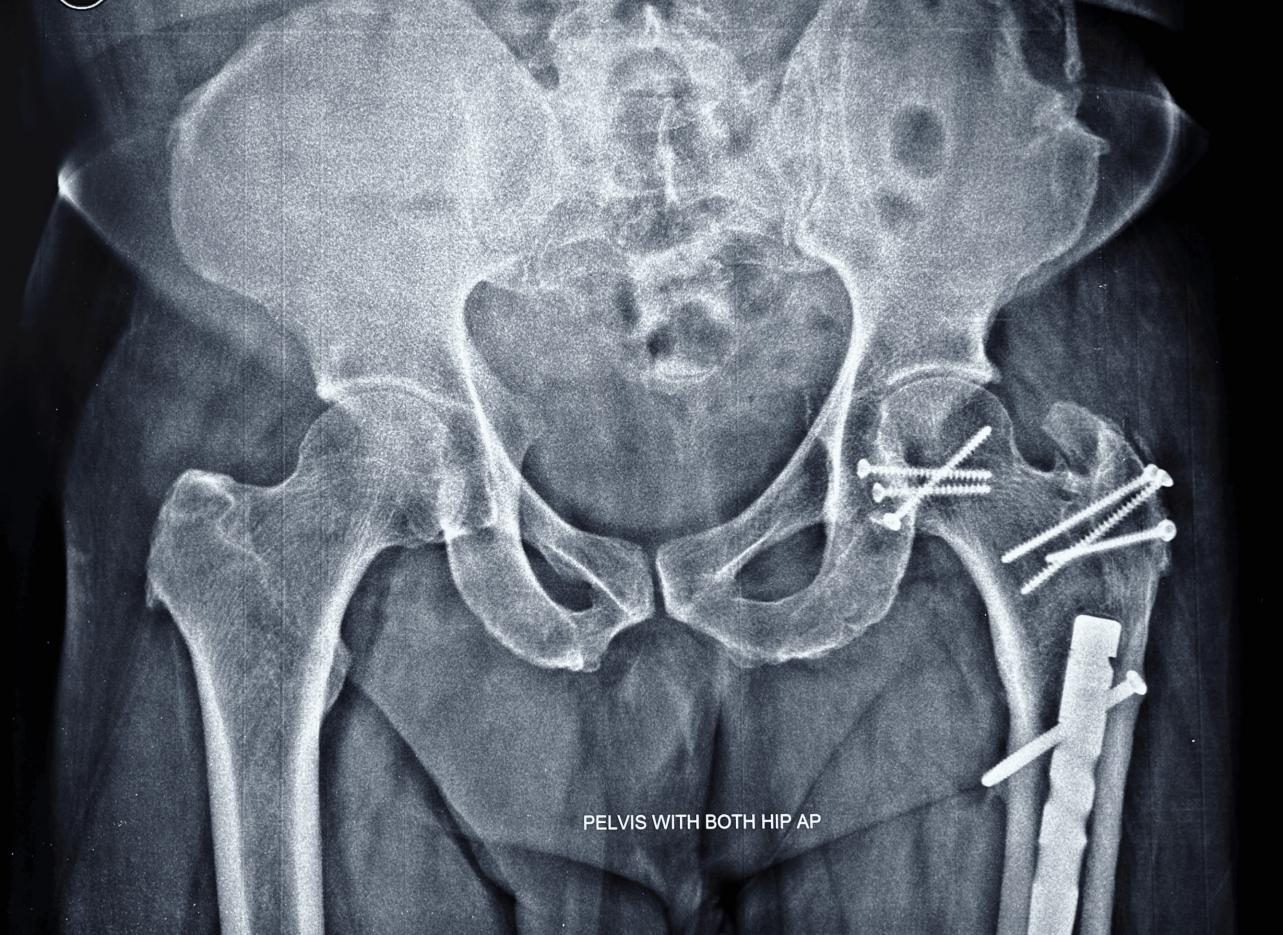
At 96 months follow-up, X-ray of both hips anteroposterior (AP) view – no signs of avascular necrosis (AVN) or post-traumatic arthritis (PTA)

**Figure 16:**
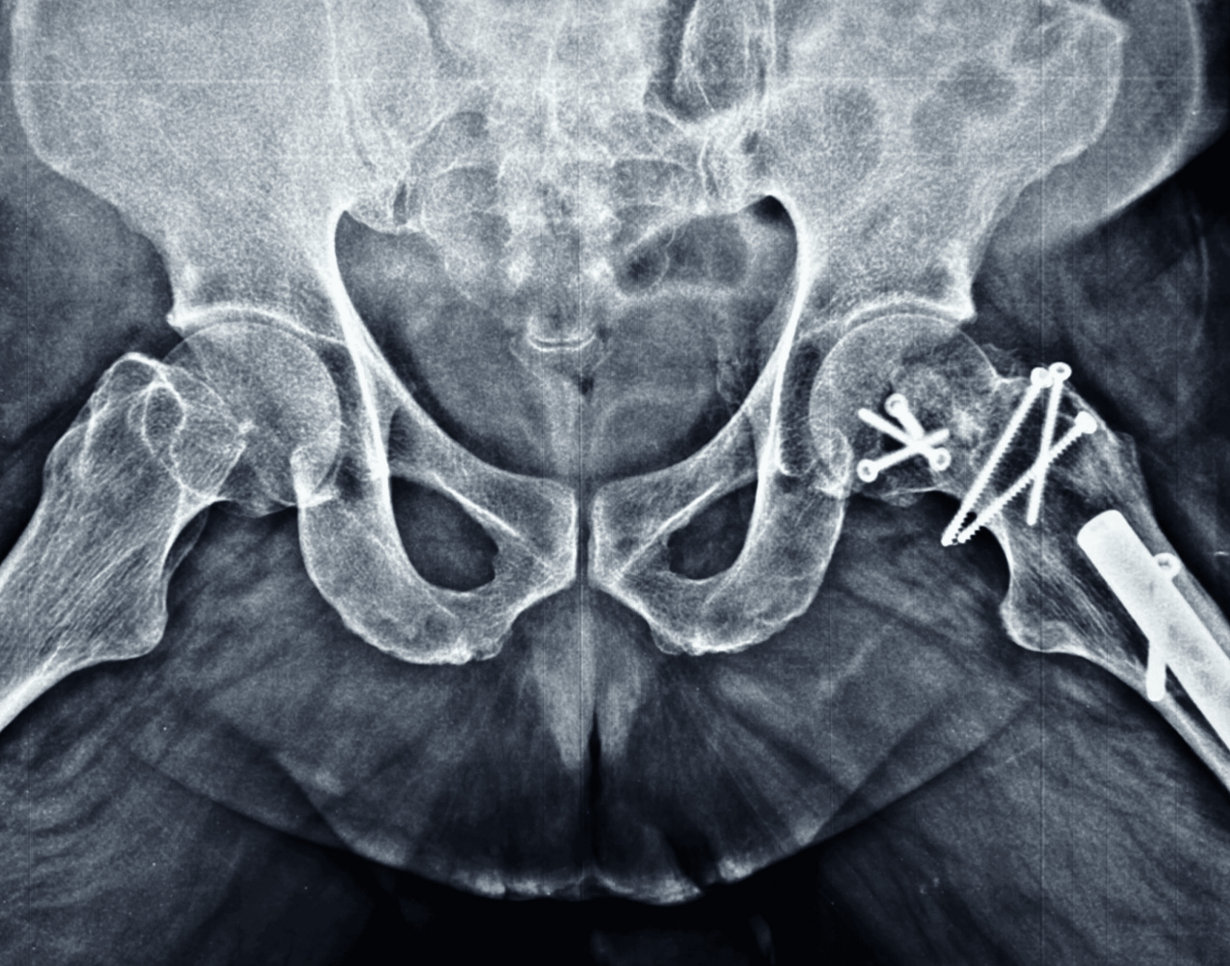
At 96 months follow-up, X-ray of both hips frog lateral view – no signs of avascular necrosis (AVN) or post-traumatic arthritis (PTA)

All had posterior acetabular labral tear, laceration which was debrided. Radiographic healing occurred at 12 weeks for both femoral head fracture and the trochanteric osteotomy site in all the cases. No case developed HO. The most notable finding in the result of this series is the nondevelopment of PTA with a follow-up period ranging from 39 months to 96 months (Figure 15, 16).

**Figure 17:**
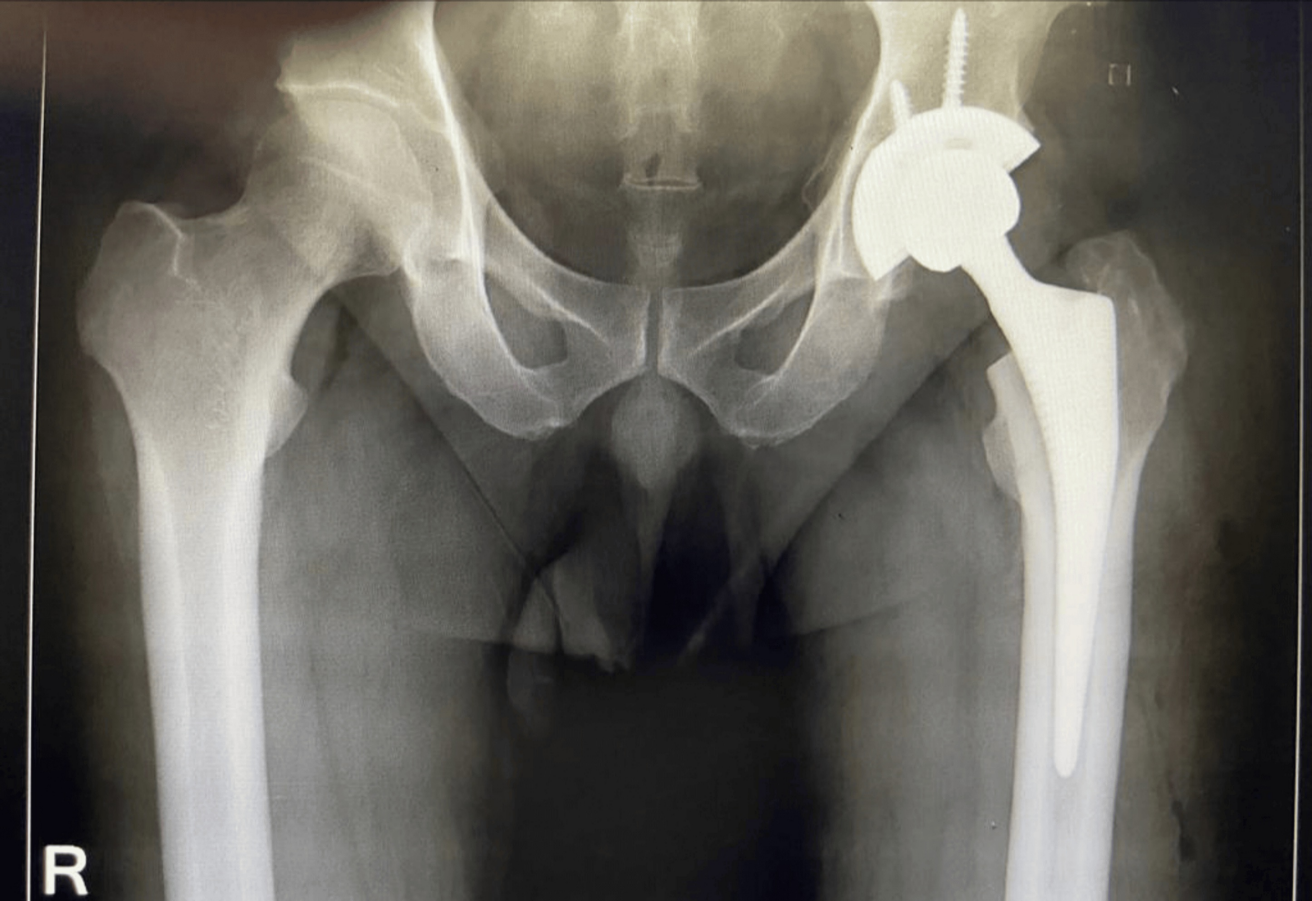
Total Hip Replacement (THR) performed

Five out of six patients returned to their usual activities except the patient who developed AVN, THR was performed (Figure 17).

Radiologically all the six cases revealed anatomical fracture head reduction and union within 12 weeks of follow-up. Trochanteric osteotomy united within 12 weeks in all.

In all cases except case no 2, the functional scores showed steady improvement until 12 weeks when all five except case no 2, achieved independent full weight-bearing mobilisation and reached their final follow-up Modified Harris Hip Score. In case no 2, pain and discomfort deteriorated as the patient developed AVN of the femoral head and a THR was performed at 19 months.

According to the Modified Harris hip score, four patients had Excellent, one had Good, and one had Poor hip score who developed AVN.

## Discussion

Controversy still exists in management and outcome of femoral head fracture among closed reduction alone, excision and ORIF using different techniques and approaches [2,3,14,23,24].

Shakya et al. in 2023 reported results analysis of outcome of femoral head fracture on 50 cases managed by various modalities of non-operative, operative by ORIF through anterior (Smith-Petersen [16]), posterior (Kocher-Langenbeck [19]), and immediate THR. Out of the complications AVN was 12%, PTA was 33%, and HO was 16%. The study concluded with aiming anatomical reduction of fragments and observed varied functional outcome and complications of treatment approaches and provided a reference for the clinical treatment to guide patient management [24].

Surgical dislocation of hip offers 360-degree visualization of acetabulum and nearly 360-degree visualization of head femur and hence an ideal exposure for working on femoral head and acetabulum. Ganz et al. reported no case of AVN in 213 hips operated on using this technique in different intra articular hip disorders over seven years [15].

We have encountered multiple minor free chondral, osteochondral, sandy particles and partly separated osteochondral and undermined articular cartilage at fracture margin. Hence these loose bodies in our opinion, may have deleterious effect on joint and may cause PTA unless the joint is exposed by Surgical dislocation of hip, all loose bodies are removed and thoroughly irrigated and fracture margins debrided properly. We also assume that the precise spherical congruent reduction with fixation of head fragment is a determining factor for the nondevelopment of PTA.

Proper handling of soft tissue, meticulous haemostasis, use of negative suction drain and prophylactic Indomethacin use may be responsible factors resulted in nil incidence of HO.

The case that developed AVN femoral head had a time interval of 52 hours between dislocation and reduction and had negative drilling test. Since femoral head fracture is a rare injury, only a few literature studies describing the technique of Ganz Surgical Dislocation of Hip in traumatic femoral head fracture exist. Our findings are similar to other studies in the literature and furthermore, PTA and HO were not encountered in our series. The time interval between hip dislocation and primary reduction, as a determining factor of AVN is reflected in our study result. The Drilling test (Bleeding sign) is one of the reliable tests to predict the viability of the femoral head in patients who undergo surgical hip dislocation. Aprato et al. reported that the sensitivity of the Drilling test or Bleeding sign is 97%, while specificity is 83% [25]. The test is easy to perform. A surgeon can decide for immediate THR with prior proper informed consent in a negative Drilling test.

Limitations of the study

There was a small number of cases, but we feel that sharing details of our experience in technique and outcome will throw some light in the direction of management of femoral head fracture.

## Conclusions

Open reduction and internal fixation of femoral head fracture by Ganz Surgical Dislocation of Hip is a viable treatment option and provides satisfactory results with low complication rate. No PTA even with a follow-up period of 96 months may be attributed or postulated to the fact that in this present technique meticulous removal of all frank and potentially loose bodies was performed and precise spherical congruent reduction of head fragment with fixation was performed. Those loose bodies, which couldn’t be assessed in post-reduction CT, may have a role in development of PTA when treated conservatively. A large analytical study with a long follow-up period is required for statistical significance. 

## References

[1] Gavaskar AS, Tummala NC (2015). Ganz surgical dislocation of the hip is a safe technique for operative treatment of Pipkin fractures. Results of a prospective trial. J Orthop Trauma.

[2] Henle P, Kloen P, Siebenrock KA (2007). Femoral head injuries: which treatment strategy can be recommended?. Injury.

[3] Mostafa MF, El-Adl W, El-Sayed MA (2014). Operative treatment of displaced Pipkin type I and II femoral head fractures. Arch Orthop Trauma Surg.

[4] Masse’ A, Aparto A, Alluto C, Favuto M, Ganz R (2015). Surgical hip dislocation is a reliable approach for treatment of femoral head Fractures. Clin Orthop Relat Res.

[5] Hosny H, Mousa S, Salama W (2022). Management of femoral head fracture by Ganz surgical dislocation of the hip. J Orthop Traumatol.

[6] Kokubo Y, Uchida K, Takeno K (2013). Dislocated intra-articular femoral head fracture associated with fracture-dislocation of the hip and acetabulum: report of 12 cases and technical notes on surgical intervention. Eur J Orthop Surg Traumatol.

[7] Stirma GA, Uliana CS, Valenza WR, Abagge M (2018). Surgical treatment of femoral head fractures through previously controlled hip luxation: four case series and literature review. Rev Bras Orthop.

[8] Birkett J (2000). Description of a dislocation of the head of femur, complicated with its fracture; with remarks by John Birkett (1815-1904) 1869. Clin Orthop Relat Res.

[9] Pipkin G (1957). Treatment of grade IV fracture-dislocation of the hip. JBJS Am.

[10] Brumback RJ, Kenzora JE, Levitt LE, Burgess AR, Poka A (1989). Fractures of the femoral head. Hip.

[11] Yoon TR, Rowe SM, Chung JY, Song EK, Jung ST, Anwar IB (2001). Clinical and radiographic outcome of femoral head fractures: 30 patients followed for 3-10 years. Acta Orthop Scand.

[12] Chiron P, Lafontan V, Reina N (2013). Fracture-dislocations of the femoral head. Orthop Traumatol Surg Res.

[13] Marsh JL, Slongo TF, Agel J (2007). Fracture and dislocation classification compendium - 2007: Orthopaedic Trauma Association classification, database and outcomes committee. J Orthop Trauma.

[14] Maximillian M (2021). Fractures of the femoral head: a narrative review. EFORT Open Rev.

[15] Ganz R (2001). Surgical dislocation of the adult hip; a technique with full access to the femoral head and acetabulum without the risk of avascular necrosis. JBJS Br.

[16] Smith-Petersen MN (1949). Approach to and exposure of the hip joint for mold arthroplasty. J Bone Joint Surg Am.

[17] Watson‐Jones R (1936). Fractures of the neck of the femur. Br J Surg.

[18] Hueter C (1883). [Fifth section: the injury and diseases of the hip joint, twenty-ninth chapter]. [Outline of Surgery].

[19] Tosounidis TH, Giannoudis VP, Kanakaris NK, Giannoudis PV (2018). The Kocher-Langenbeck approach: state of the art. JBJS Essent Surg Tech.

[20] Gibson A (1950). Posterior exposure of the hip joint. J Bone Joint Surg Br.

[21] Ludloff K (1913). The open reduction of the congenital hip dislocation by an anterior incision. JBJS.

[22] Ferguson AB Jr (1973). Primary open reduction of congenital dislocation of the hip using a median adductor approach. J Bone Joint Surg Am.

[23] Peng SH, Wu CC, Yu YH, Lee PC, Chou YC, Yeh WL (2019). Surgical treatment of femoral head fractures. Biomed.

[24] Shakya S, Chen J, Sun J, Xiang Z (2023). Management and outcome of patients with femoral head fractures: the mid-term follow-up with injuries and associated prognostic factors. BMC Musculoskelet Disord.

[25] Aprato A, Bonani A, Giachino M, Favuto M, Atzori F, Masse’ A (20141). Can we predict femoral head vitality during surgical hip dislocation?. J Hip Preserv Surg.

